# Enhancing Radiation Shielding Properties of Pharmaceutical Polymers Through Zinc Oxide Incorporation: A Study on Gamma Energy Attenuation

**DOI:** 10.3390/polym17212859

**Published:** 2025-10-27

**Authors:** Mohammad W. Marashdeh, Afnan Alsalman, Muthanna Abdulkarim

**Affiliations:** 1 Department of Physics, College of Sciences, Imam Mohammad Ibn Saud Islamic University (IMSIU), P.O. Box 90950, Riyadh 11623, Saudi Arabia; 433024187@sm.imamu.edu.sa; 2 Department of Pharmaceutical Sciences, College of Pharmacy, Alfaisal University, P.O. Box 50927, Riyadh 11533, Saudi Arabia; malbaldawi@alfaisal.edu

**Keywords:** Benecel K4M, zinc oxide composites, gamma radiation shielding, pharmaceutical polymers, radiation protection efficiency, mean free path

## Abstract

An investigation was carried out to improve the gamma radiation shielding properties of Benecel K4M pharmaceutical polymer using zinc oxide (ZnO) at concentrations from 0 to 6 wt.%. Compressed composite tablet samples were prepared and tested in the range of photon energies 59.5 to 1332 keV for the assessment of various shielding parameters, including linear attenuation coefficient, radiation protection efficiency (RPE), and mean free path (MFP). As the ZnO content increased, the attenuation properties of the material showed improved shielding behavior, which was attributed to its high density and atomic number. At 59.9 keV, RPE increased from 6.9% for the pure polymer to 12.2% for the 6 wt.% composite, whereas MFP decreased from 13.9 cm to 7.6 cm. The results indicate that ZnO addition significantly enhances the shielding efficiency of Benecel K4M, demonstrating that ZnO can serve as a lightweight and non-toxic alternative to heavy-metal-based materials for pharmaceutical protection in radiation-rich environments.

## 1. Introduction

As ionizing radiation is now being used in medical diagnosis, therapeutic, and industrial applications and other fields, the need for reliable and effective radiation protection has become increasingly important, especially for drug final products (medicines). Exposure of drugs to ionizing radiations such as gamma and X-rays can result in drug degradation, production of harmful toxic products, the resultant loss in drug efficiency, and potential risk to patients [1,2]. This problem is of high concern in medical facilities with radiation equipment, radiation sterilization of medical devices, and during air transport of medicines where there are significantly higher levels of radiation. Thus, there is a need to create effective shielding materials to ensure the stability and safety of the pharmaceuticals under such conditions.

The effectiveness of a radiation shield is determined by its chemical and micro-structural characteristics. Elements with high atomic numbers, such as bismuth or heavy metal oxides, can improve the attenuation when mixed with low-attenuation materials, making them excellent candidates [3,4]. More importantly, the current aim is to identify green shielding components (e.g., naturally occurring or biodegradable materials) to replace toxic alternatives in pharmaceutical and materials industries [5,6]. For example, traditional shielding materials like lead are being phased out because of their toxicity and environmental impact.

On the other hand, the light weight, ease of processing, and non-toxic characteristics of polymers and polymer-based composites make them an excellent substitute. Composites blended with inorganic fillers can provide high radiation attenuation without losing mechanical flexibility [7,8]. Composite systems consisting of organic matrices with inorganic additives show a synergistic effect that improves shielding performance [9]. Various polymers such as high-density polyethylene (HDPE) [10], polycarbonate (PC) [11], epoxy resins [12], polyurethane (PU) [13], and polymethyl methacrylate (PMMA) [14] have been studied for gamma-ray shielding at different energy ranges.

In pharmaceutical applications, polymers are extensively used in drug delivery systems due to their high degradability and not being harmful to living tissue, while regulating drug release. As noted by Debotton and Dahan [15], oral solid dosage forms serve as important advanced drug delivery systems for medicines and can be modified into advanced delivery technologies for biologics and other sensitive agents. According to Onesto et al. [16], sustained-release delivery of labile biomolecules can be achieved using biodegradable microneedles fabricated from poly(lactic-co-glycolic acid) (PLGA). Similarly, natural polymer hydrogels such as gelatin and dextran have been utilized for topical drug delivery applications, while polysaccharide-based carriers, including chitosan and alginate, have been employed for systemic drug delivery and encapsulation [17,18]. These polymer systems are ideal candidates to simultaneously provide drug delivery and radiation shielding functions.

Hypromellose (hydroxypropyl methylcellulose, HPMC) and its derivative Benecel K-4M are highly used excipients in pharmaceutical formulation for their ability to form hydrophilic matrices in sustained drug delivery systems. In conventional applications, this gel-forming property enables a sustained hydration and swelling of tablet matrix and drug diffusion. When applied as a coating or incorporated into pharmaceutical particles and tablets, HPMC forms a thick, cohesive layer providing mechanical integrity to the delivery system. Such uniform protective shells around solid dosage forms or pharmaceutical particulates could offer a dual benefit of controlled release and enhanced shielding capacity. The addition of zinc oxide (ZnO) to various polymeric or composite materials has been gaining attention because of its advanced structural, thermal, and radiation-shielding properties. Zinc oxide (ZnO) has a high atomic number along with great chemical stability. Further, it interacts well with electromagnetic radiation. It is a multifunctional metal oxide. Therefore, it serves as a suitable additive for enhancing attenuation performance in polymer-based systems. Recent studies indicate the broad application of ZnO-based nanocomposites in various fields such as magnetic materials, gas sensors, and radiation-absorbing systems [19,20,21]. The embedding of ZnO within the polymeric matrix enhances shielding behavior. This is due to the high atomic number and density of ZnO, which enhance the attenuation efficiency through the processes of photoelectric absorption and scattering of the photons [22,23]. Earlier studies have shown that ZnO-containing composites have better gamma shielding performance without losing the structural performance required for application [24,25,26,27].

The factor that determines the shielding efficiency is the concentration of ZnO in the polymer matrices. Higher loading of filler material like ZnO generally increases attenuation; however, this may compromise the physical and mechanical properties of the composites [28,29]. More nanocomposite systems support the argument that one carefully engineered polymer–inorganic interface can achieve great improvement in radiation shielding performance [30,31].

Motivated by these observations, this paper is devoted to developing polymer composite of Benecel K-4M filled with different concentrations of ZnO. The shielding of radiation is evaluated in a systematic way in terms of energy of photon ranging from 59.5 to 1332 keV. The purpose of this study is to address the research gap on attenuation coefficients of pharmaceutical polymer–ZnO composites and to study the effect of filler concentration on important radiological parameters, such as mean free path (MFP) and radiation protection efficiency (RPE).

## 2. Materials and Method

### 2.1. Fabrication of Benecel K-4M Samples

Materials: Zinc oxide was purchased from Intermediate Chemicals Company-ARABIAN ZINC, Riyadh, Saudi Arabia. Benecel K-4M (Ashland, OR, USA) was a gift from Brenntag Saudi Arabia Ltd. Magnesium stearate (Lobachemie, India) was purchased from a local supplier.

**Figure 1:**
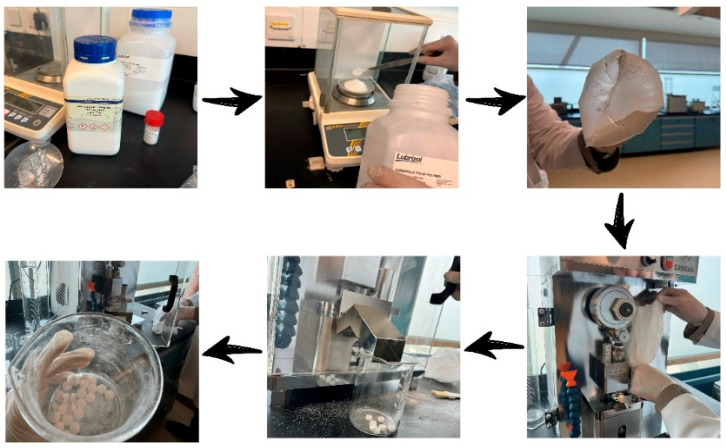
Steps of fabrication of tablet samples.

All materials were accurately weighed using an analytical balance (Kern, & Sohn GmbH, Balingen, Germany). As shown in Figure 1, the polymer and zinc oxide powders were mixed inside a securely sealed double plastic bag. To achieve uniform powder distribution, mixing was carried out for 5 min by continuous horizontal agitation. Subsequently, before the compression, magnesium stearate was added into the plastic bag and stirred with the rest of the ingredients slightly to maintain its function as a surface lubricant rather than allowing complete distribution throughout the mixture. Following equipment preparation verification, the final blend was loaded into the feeder of the single-punch tablet compression machine (Erweka Tablet Press-1, Erweka GmbH, Langen, Germany). The tablets were given the codes S0, S2, S4, and S6, with each formulation containing zinc oxide being 0%, 2%, 4%, and 6%, respectively, as shown in Table 1. Due to the hygroscopic nature and low compressibility properties of zinc oxide, the maximum ratio for zinc oxide was 6%. The ZnO amount used (≤6 wt%) is safe and falls within the limits of various pharmaceutical, topical, and dietary formulations. At such levels there is less leaching or toxicity. Earlier studies revealed that ZnO was toxic at higher concentrations. This adverse effect is primarily due to the dissolved zinc ions and not the ZnO nanoparticles. The research of Wu et al. [32], Yan et al. [33], Sahu et al. [34], and Waalewijn-Kool et al. [35] agrees that a lower concentration of ZnO shows superior biocompatibility and a lesser toxic effect. Having ZnO content below 6 wt% will keep the composites in the safe and acceptable exposure limits for proposed pharmaceutical and packaging products. The mixing and compression processes were repeated with different percentages of zinc, taking into account cleaning the tablet compression machine between each batch to ensure that there is no remaining powder from the previous sample. Every mixing and compression step was made in sealed conditions. The measured values of prepared tablets were carried out immediately after preparation to minimize any possible effect of humidity on either density or shielding performance. The density of the Benecel K4M tablet samples was determined using a caliper to measure and an electronic balance to determine mass. Measurements were repeated and results were recorded in similar units (grams and micrometers).

### 2.2. Linear and Mass Attenuation Coefficient Measurements

**Figure 2:**
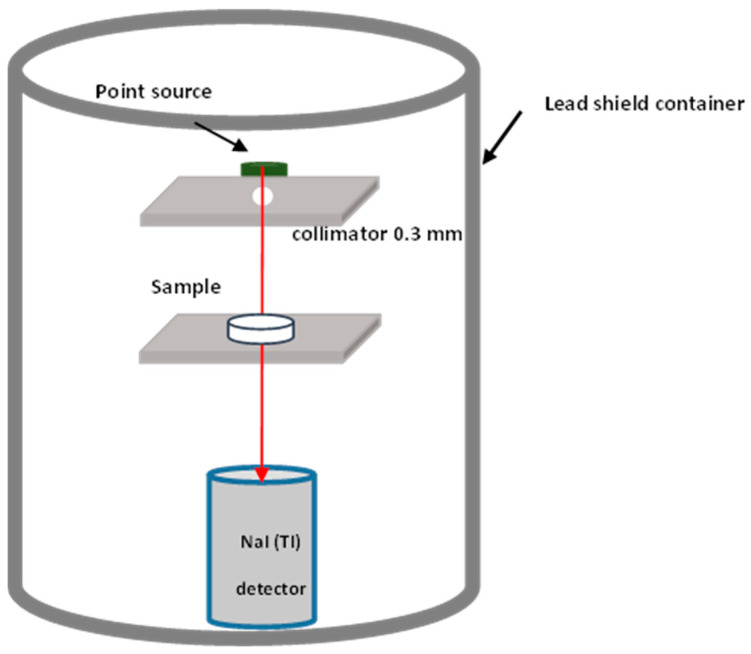
Schematic of the experimental setup.

We irradiated the samples with three standard point sources, namely, Americium-241 ^214^Am (59.5 keV), Caesium-137 ^137^Cs (661.6 keV), and Cobalt-60 ^60^Co (1173.2 and 1332.5 keV). A sodium iodide (NaI(Tl)) scintillation detector was used to measure the samples’ energy intensity. The measurements were carried out inside a lead shielding container to protect against background radiation and scattering. The 256 channel Multi-Channel Analyzer (MCA) was used for analyzing and detecting gamma spectra with detail spectrum information. All samples have been subjected to radiation for 1800 s. Collimators with 0.3 cm diameter were placed in front of the source directly facing the detector, as shown in the setup in Figure 2. The distance between a point source and the sample was 4 cm, while that between a point source and the detector was 21.6 cm. After the source was mounted, the initial gamma-ray intensity, recorded as I_0_, was measured. Subsequently, gamma-ray counts were recorded for individual tablet samples of different thicknesses. The experiment was repeated three times for each reading. To ensure unbiased observation, the relative counts (I/I_0_) computed for various thickness values of the samples were analyzed.

### 2.3. Theory

When a gamma-photon beam passes through a material, its intensity decreases due to interactions such as photoelectric absorption, Compton scattering, and pair production. This attenuation process follows the Beer–Lambert law [36]:

(1) $$I=I_{0}{e\;}^{-µx}$$

where *x* is the material thickness (cm), I_0_ is the initial beam intensity, I is the transmitted intensity, and µ is the linear attenuation coefficient (cm^−1^) [37].

To eliminate the effect of density, the mass attenuation coefficient (μ/*ρ*) is used, expressed as:

(2) $$\mu /\rho =\sum_{i}{\left(\mu /\rho \right)}_{i}w_{i}$$

where (μ/*ρ*)_i_ represents the mass attenuation coefficient of the *i*th element and w_i_ is its weight fraction. The error of ∆ μ⁄*ρ* measurement is calculated using Equation (3):

(3) $$\frac{∆\mu /\rho }{\mu /\rho }=\sqrt{{\left(\frac{∆m_{\rho }}{m_{\rho }}\right)}^{2}+{\left(\frac{∆I}{I}\right)}^{2}+{\left(\frac{∆I_{0}}{I_{0}}\right)}^{2}}$$

where ∆m*_ρ_*, ∆I_0_, and ∆I are the error of the mass density, unattenuated photon intensity, and attenuated photon intensity, respectively.

Another related parameter, the mean free path (MFP), represents the average distance traveled by photons between two interactions and is given by [38]:


(4) MFP=1μ

Radiation Protection Efficiency (RPE) quantifies the shielding capability of a material and is derived from the linear attenuation coefficient as follows [39]:

(5) $$RPE=\left(1-e^{-\mu }\right)\times 100\left(\%\right)$$

## 3. Results and Discussion

### 3.1. Linear and Mass Attenuation Coefficient Measurement

This study shows that incorporating zinc oxide (ZnO) into Benecel K4M improves its gamma radiation shielding capabilities. The densities of the prepared samples steadily increased with increasing ZnO contents. The density of the pure polymer S0 was found to be 0.493 g/cm^3^ (Table 1), while that of the 6 wt.% ZnO composite S6 was 0.678 g/cm^3^. The increase in density directly improved the interaction probability of photons. This is due to the fact that dense material offers higher attenuation. This property is useful for pharmaceutical applications that need radiation protection and lightweight material.

In Table 2 the values of linear attenuation coefficient (µ) are strongly influenced by photon energies and filler concentration. At 59.5 keV, the experimental µ rose from 0.072 cm^−1^ for S0 to 0.131 cm^−1^ for S6, indicating an improvement of 82% due to addition of ZnO. The theoretical values determined by XCOM were consistently higher (for example, 0.096 cm^−1^ for S0 and 0.183 cm^−1^ for S6) with a difference range of 21–29% at this energy. The difference arises because, at low energies, the photoelectric effect dominates as this effect is very sensitive to the elementary composition and density assumptions made in the theoretical calculations. Variations in sample homogeneity and dispersion of ZnO can cause such large differences between experimental and theoretical values.

At intermediate energies, like 661.6 keV, where Compton scattering is the dominating interaction, the attenuation coefficients become smaller but continue to reflect the effect of ZnO loading. Experimental values of µ were found to be 0.035 cm^−1^ (S0) to 0.066 cm^−1^ (S6), with theoretical predictions of 0.041 cm^−1^ and 0.056 cm^−1^, as shown in Table 2. Some samples (like S4) were found to have experimental values greater than theoretical values (for example, S4 0.056 cm^−1^ vs. 0.052 cm^−1^) as strong scattering of photons by uniformly dispersed particles of ZnO in the matrix might not be reflected theoretically.

A comparable trend occurred at higher energies (1173 to 1332 keV), where µ further dropped due to Compton scattering and pair production jointly dominating. The experimental values for S0 and S6 at 1332 keV were 0.027 cm^−1^ and 0.038 cm^−1^, respectively. These values closely agreed with the theoretical values of 0.029–0.040 cm^−1^ and diverged from them only 3–5%. The close agreement is evidence that the experiment and the theory are reliable at high energies where the interaction mechanism is not sensitive to atomic number changes.

**Figure 3:**
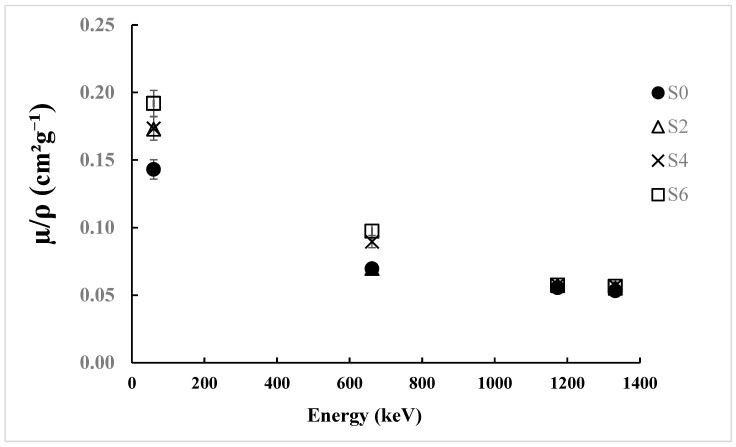
Experimental mass attenuation coefficients for Benecel K4M tablet samples.

The observations are backed by the data related to attenuation coefficient. The experimental µ/*ρ* values at 59.5 keV were 0.143 cm^2^/g for S0 and 0.192 cm^2^/g for S6, as shown in Table 3. Their theoretical values were 0.194–0.270 cm^2^/g, which gave discrepancies of 21–29%. However, at 1332 keV, the difference narrowed down to 4–5%, with experimental values (0.053–0.057 cm^2^/g) matching well with theoretical (0.060–0.059 cm^2^/g), as shown in Figure 3. At higher energies, the convergence implies that atomic number plays a lesser role in determining the interaction probability, which is consistent with the transition from photoelectric effect to Compton scattering.

With the increasing quantity of ZnO, the shielding efficiency improves progressively at all energy levels. The enhancement in µ/*ρ* values at lower energies (maximum enhancement of 34% for S6 over S0 at 59.5 keV) reveals the high effectiveness of ZnO in the photoelectric absorption process. On the other hand, the enhancements in µ/*ρ* values at higher energies indicate the limited yet existing effectiveness of ZnO under Compton scattering condition. The results are consistent with those demonstrated by Alsayed et al. [36], which showed similar behavior of ZnO-based composites, indicating that the optimal filler concentration can provide desired shielding effectiveness.

In other words, the experimental results showed the accuracy of theoretical calculations and that the observed differences are due to the variation in composite density and dispersion of ZnO. The composites containing ZnO exhibited a mechanically strong correlation between the density and attenuation properties. Therefore, these composites can have potential applications in pharmaceutical packaging and drug delivery systems that may be exposed to ionizing radiation during processing, storage, or transport. The lightweight and biocompatible properties of Benecel K4M ensure that these improved shielding properties do not hinder the practical applications of this material in the medical sector.

To evaluate the shielding performance of the fabricated samples of Benecel K-4M/ZnO composites, three polymer systems from earlier studies are chosen. These include HDPE, PVA, and PVP as shown in Table 4. These materials were selected based on their composition and established use in the radiation-shielding literature [40,41] rather than on structural similarity. The choice of the high-density polyethylene (HDPE) reinforced with ZnO was made because it is one of the most widely studied in terms of the polymer-based shielding and, importantly, the ZnO concentrations (2–6 wt.%) are similar to those used in the present study. Thereby, the density of HDPE-based composites (0.96–1.14 g cm^−3^) is much higher than that of the present Benecel samples (0.49–0.68 g cm^−3^). However, the measured (μ/*ρ*) of the Benecel composites are within a comparable range, particularly at lower energies. For example, at 661.6 keV, the S6 (6 wt.% ZnO) sample recorded μ/*ρ* = 0.097 cm^2^ g^−1^, which is a value that is slightly higher than 0.084 cm^2^ g^−1^ for 6 wt.% ZnO–HDPE. Even though the density of the Benecel matrix is lower because of the embedded ZnO nanoparticles, photon interaction occurred, which caused the reasonable enhancement of attenuation performance.

On the other hand, polyvinyl alcohol (PVA) and polyvinyl pyrrolidone (PVP), due to their pharmaceutical relevance and hydrophilic nature [42], which is similar to Benecel K-4M, were selected for comparison. The three are biocompatible water-soluble polymers, and they are commonly used in pharmaceutical formulations, making them suitable for studies on radiation shielding performance. The μ/*ρ* values for PVA and PVP at 661 keV were 0.0870 and 0.0861 cm^2^ g^−1^, respectively [41]. The Benecel/ZnO samples present showed μ/*ρ* values in the range of 0.070 to 0.097 cm^2^ g^−1^ depending on the ZnO loadings. Thus, it has a similar attenuation behavior. The small difference between the measured value and that found in the literature suggests that the dominant role played by ZnO loading is in enhancing photon attenuation. Differences in the host polymer affect mainly density and dispersion efficiency. The μ/*ρ* of the Benecel samples S4 and S6 increased slightly due to the scattering and absorption process. The ZnO particles showed uniformity in the cellulose-based sample.

Compared to HDPE, PVA, and PVP systems, the fabricated Benecel K-4M/ZnO composites have been confirmed to show reasonable shielding performance. This finding is despite the lower density of Benecel composites, making them excellent candidates for lightweight and biocompatible radiation protection. Benecel K-4M, unlike HDPE, which is not biodegradable, is cellulose-based and is an excipient, which is already approved for use in drug formulation. This material’s flexibility and safety, coupled with its ability to integrate ZnO nanoparticles, show its relevance in a pharmaceutical and biomedical context. Such use is possible for protective packaging, controlled-release coating, and localized shielding in radiopharmaceutical settings. As a result, the damping effectiveness of the Benecel-structured composites is not as good as that of the denser polymers; nevertheless, their low toxicity, low weight, and process ability provide a valuable compromise between efficiency and safety. Thus, they are appealing materials for pharmaceutical-grade radiation shielding purposes.

### 3.2. Mean Free Path (MFP)

The mean free path (MFP) results of RPE for the ZnO tablet samples are presented from photon energy of 59.9 to 1332 keV. They indicate the changes due to the combined effect of energy and ZnO concentrations on the attenuation of photons. MFP is the average distance that a photon transports before interacting with the material. Thus, the lower the MFP, the stronger the attenuation.

**Figure 4:**
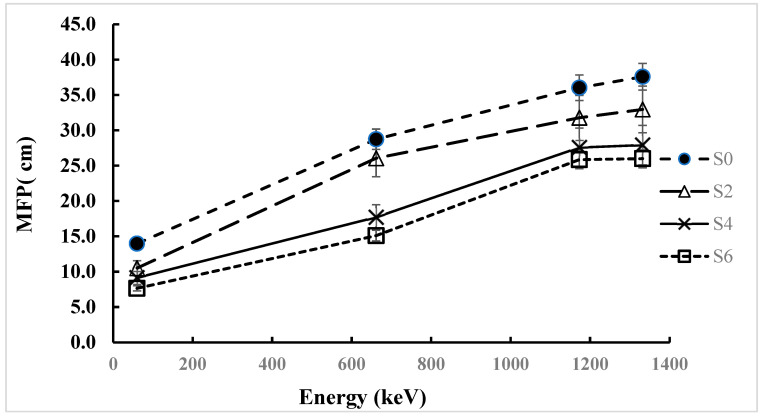
Experimental mean free path for Benecel K4M tablet samples.

As shown in Figure 4, the MFP values at 59.9 keV were the lowest for all the samples due to the photoelectric effect. Benecel K-4M only (S0) recorded an MFP value of 13.977 cm, while the 6 wt.% ZnO composite polymer (S6) achieved a value of 7.663 cm, which indicates a 45% reduction in value. Correspondingly, intermediate samples S2 and S4 recorded 10.513 cm and 9.153 cm, respectively, showing a clear decrease with increasing ZnO content.

With increasing photon energy, the MFP values significantly increased due to weaker interactions at those energies. At an energy of 661.6 keV, the MFP increased to 28.736 cm for S0 and reduced to 15.099 cm for S6. Such a drop indicates that, even at Compton-dominated energies, the free path length is decreased by approximately 50% due to the incorporation of ZnO. Following a comparable pattern at both 1173 keV and 1332 keV, the MFPs of S0 were recorded as 36.022 cm and 37.579 cm, whereas S6 exhibited lower MFPs of 25.851 cm and 25.993 cm, respectively (Figure 4).

As the ZnO concentration increases, the MFP is steadily reduced, indicating that the high-Zn fillers enhance the attenuation performance. Although the overall MFP increased with increased photon energy, the relative enhancement of MFP by ZnO was significant in the entire energy range, indicating the composite was suitable for multi-functional shielding application.

### 3.3. Radiation Protection Efficiency (RPE)

The parameter that quantifies how effectively a shielding material attenuates radiation intensity is termed as RPE. The RPE results show similar findings to [43], where higher radiation protection efficiencies were observed at low energies and increasing efficiencies were observed with higher ZnO concentrations.

**Figure 5:**
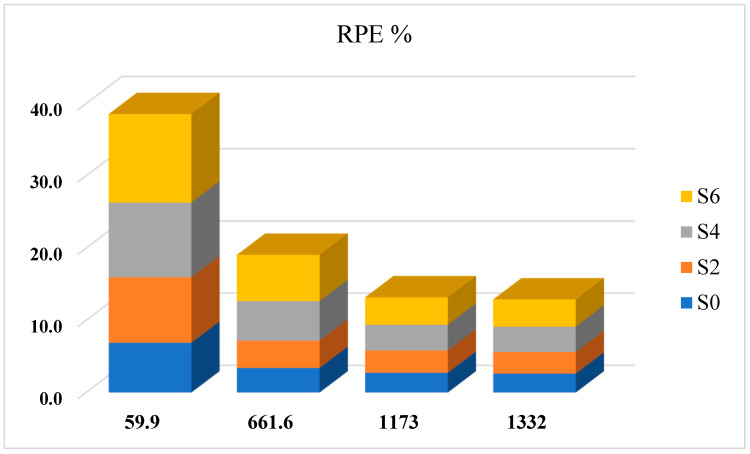
RPE variation for the Benecel K4M tablet samples with energy and additive ZnO quantity.

As shown in Figure 5, at 59.9 keV, RPE values were obtained in the range of 6.905% for S0 to 12.235% for S3. The results for S2 and S4 were 9.074% and 10.349%, respectively, which show the increased efficiency with increased ZnO load.

As energy increases, RPE values decrease because Compton scattering starts to dominate and photoelectric contributions reduce. The RPE value at 661.6 keV for S0 was 3.420% and for S6 improved to 6.409%. At 1173 keV, RPE varies from 2.738 to 3.794% and at 1332 keV RPE varies from 2.626 to 3.774%.

The efficiency may be lower at the highest energies, but composites loaded with ZnO always outperform the pure polymer, thus showing a much superior attenuation property.

The rise in RPE at all energies shows that ZnO is an effective filler for pharmaceutical polymers. A composite of low polymer density with high-Z additive provides a stable solution for lightweight shield materials under any gamma energy. This property is especially important for pharmaceutical packaging and drug delivery systems, which require biocompatibility and sufficient radiation protection.

### 3.4. Effective Atomic Number (Z_eff_) and Electron Density (N_eff_)

**Figure 6:**
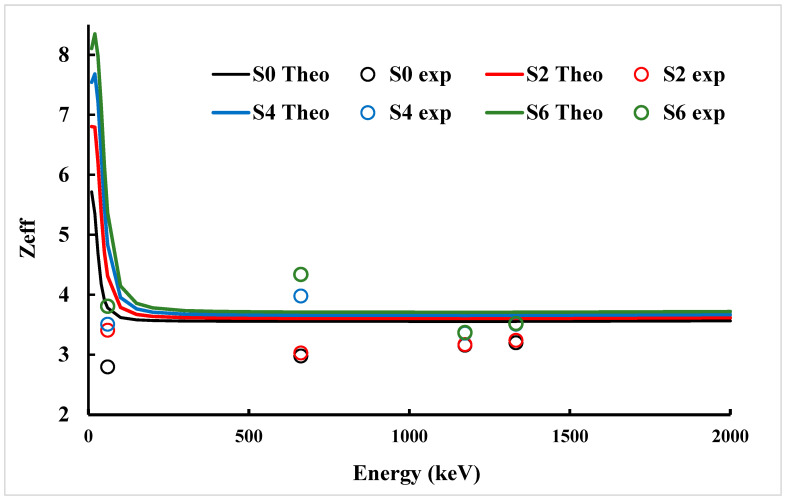
The effective atomic number as a function of photon energy for studied Benecel K4M tablet samples.

The changes in the Effective Atomic Number (Z_eff_) with gamma photon energy and with different concentration of ZnO provide strong evidence that the filler concentration plays an important role in enhancing the gamma-ray interaction in Benecel K4M composites. Figure 6 shows Z_eff_ values that increased consistently with higher ZnO loading at all energies in this study. As noted in Figure 6, for S0 (pure polymer), Z_eff_ at 10 keV was 5.72 and increased to 8.10 for S6 (6 wt.% ZnO), while, at 59.9 keV, the values increased from 3.78 to 5.37. Experimental results showed a similar trend but were lower than theoretical values. Z_eff_ at 59.9 keV increased about 36% from 2.80 (S0) to 3.81 (S6). The reason for the difference between the experimental and theoretical results is more significant at lower energies and is attributed to variations in the density and filler homogeneity, which greatly affect the photoelectric interaction. The gap is considerably reduced at very high energy (1332 keV). For S0, experimental and theoretical values were 3.2 versus 3.56 and, for S6, they were 3.52 versus 3.71. This shows the atomic number has less effect on Compton scattering temperature.

**Figure 7:**
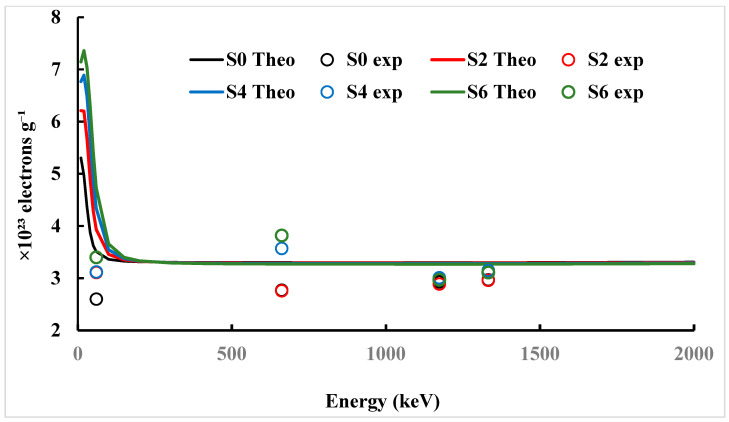
The electron density (N_eff_) as a function of photon energy for studied Benecel K4M tablet samples.

Figure 7 shows a similar trend for the electron density (N_eff_). The theoretical Neff values increased from 5.31 × 10^23^ electrons/g (S0) to 7.14 × 10^23^ electrons/g (S6) at 10 keV, which means more ZnO provides more electrons for the incident photons to react with. Experimental results at 59.9 keV also showed improvement from 2.6 for S0 to 3.4 for S6, resembling a 30% improvement. As the energy increased, the differences between the theoretical and the experimental N_eff_ values started to converge. The notable difference between the experimental and theoretical values at lower energies can be related to micro-porosity, the varying distribution of non-octagonal species such as ZnO, and surface irregularities that reduce the actual electron density compared to the ideal homogeneous model. The values at 1332 keV converged to around 2.97 to 3.11 experimentally and 3.30 to 3.27 theoretically. The closeness of these values indicates that, at high energies, Compton scattering dominates and the calculated electron density is less impacted by the atomic number. As a whole, the enhancements in Z_eff_ and Neff show that adding ZnO improves the polymer matrix’s ability to interact with photons and would be of significant advantage for pharmaceutical radiation shielding.

## 4. Conclusions

Incorporating zinc oxide (ZnO) into Benecel K4M exhibited larger diminution of gamma radiation over a wide photon energy range of 59.5–1332 keV. The shielding parameters were improved by increasing ZnO concentration from 0 to 6 wt.%. At a low energy dominated by the photoelectric effect, the linear attenuation coefficients and radiation protection efficiencies were significantly enhanced; for example, RPE increased from 6.9% to 12.2% at 59.9 keV. The mean free path values also reduced significantly from 13.9 cm at 0% ZnO to 7.6 cm for 6 wt.% ZnO composite at the same energy, showing a greater photon interacting efficiency. The findings indicate that a relatively small load of ZnO can significantly improve the shielding ability of pharmaceutical-grade polymer without adding much weight.

The detection of effective atomic number (Z_eff_) and the electron density (N_eff_) also confirmed this finding. Z_eff_ values increased from 5.716 to 8.104 at 59.5 keV and from 3.7818 to 5.3662 at 10 keV. N_eff_ also showed similar behavior, where it increased from 5.3056 × 10^23^ electrons/g to 7.1442 × 10^23^ electrons/g with ZnO incorporation. The consistent agreement between experimental and theoretical data at higher energy confirmed the reliability of the composite design and measuring method. The results above clearly show that ZnO-loaded Benecel K4M has potential to be used as safe, lightweight, and effective alternative shielding material for heavy metals in the radiation field for manufacturing, storage, and transport of pharmaceutical products.

## Figures and Tables

**Table 1:** Formulations of Benecel k4M samples with different additives and density measurmnet.

Composites	mg/Tablets			Concentration (wt.%)			Density (g/cm^3^)
	Benecel K-4m	Zinc Oxide	Magnesium Stearate	Benecel K-4m	Zinc Oxide	Magnesium Stearate	
S0	544.0	0.0	5.5	99.0	0.0	1.0	0.493
S2	533.5	11.0	5.5	97.0	2.0	1.0	0.550
S4	522.5	22.0	5.5	95.0	4.0	1.0	0.626
S6	511.5	33.0	5.5	93.0	6.0	1.0	0.678

**Table 2:** Experimental and theoretical (XCOM) linear attenuation coefficient (cm^−1^) of fabricated Benecel K4M samples.

Energy (keV)	So			S2			S4			S6		
	The.	Exp.	∆	The.	Exp.	∆	The.	Exp.	∆	The.	Exp.	∆
59.5	0.096	0.072	−25.32	0.121	0.095	−21.28	0.153	0.109	−28.79	0.183	0.131	−28.82
661.6	0.041	0.035	−15.69	0.046	0.038	−16.45	0.052	0.056	8.27	0.056	0.066	17.50
1173	0.031	0.028	−11.62	0.035	0.031	−9.97	0.040	0.036	−8.56	0.043	0.039	−9.75
1332	0.029	0.027	−9.56	0.033	0.030	−7.34	0.037	0.036	−3.61	0.040	0.038	−4.19

**Table 3:** Mass attenuation coefficient (cm^2^/g) of fabricated Benecel K4M samples.

Energy (keV)	S0			S2			S4			S6		
	The.	Exp.	∆	The.	Exp.	∆	The.	Exp.	∆	The.	Exp.	∆
59.5	0.194	0.143 ± 0.003	−26.37	0.220	0.173 ± 0.001	−21.28	0.245	0.173 ± 0.001	−29.24	0.270	0.192 ± 0.001	−29.03
661.6	0.084	0.070 ± 0.004	−16.87	0.084	0.070 ± 0.002	−16.45	0.083	0.090 ± 0.001	7.58	0.083	0.097 ± 0.001	17.16
1173	0.064	0.056 ± 0.003	−12.86	0.064	0.057 ± 0.002	−9.97	0.063	0.058 ± 0.001	−9.14	0.063	0.057 ± 0.002	−9.11
1332	0.060	0.053 ± 0.003	−10.83	0.060	0.055 ± 0.002	−7.34	0.059	0.057 ± 0.001	−4.22	0.059	0.057 ± 0.002	−4.47

**Table 4:** Comparison of mass attenuation coefficient (cm^2^/g) and measured density (g/cm^3^) of fabricated Benecel K4M samples and other studies at 661.6, 1173, and 1332 keV.

Materials	*µ/ρ* (cm^2^/g)				Density (g/cm^3^)
	Study Theme	661.6 keV	1173 keV	1332 keV	
S0 (current work)	Experimental	0.070	0.056	0.053	0.493
S2 (current work)	Experimental	0.070	0.057	0.055	0.550
S4 (current work)	Experimental	0.090	0.058	0.057	0.626
S6 (current work)	Experimental	0.097	0.057	0.057	0.678
HDPE (high density polyethylene) [40]	Experimental	0.085	0.066	0.063	0.960
2% ZnO—HDPE [40]	Experimental	0.0848	0.0654	0.065	1.030
4% ZnO—HDPE [40]	Experimental	0.0843	0.0653	0.066	1.070
6% ZnO—HDPE [40]	Experimental	0.084	0.067	0.067	1.140
Polyvinylalcohol (PVA) [41]	Calculated	0.0870	0.0626	0.0576	1.190
Polyvinylpyrrolidone (PVP) [41]	Calculated	0.0862	0.0620	0.0571	1.200

## Data Availability

Data are contained within the article.
